# Electrical Control of Magnetic Resonance in Phase Change Materials

**DOI:** 10.1021/acs.nanolett.4c02697

**Published:** 2024-09-04

**Authors:** Tian-Yue Chen, Haowen Ren, Nareg Ghazikhanian, Ralph El Hage, Dayne Y. Sasaki, Pavel Salev, Yayoi Takamura, Ivan K. Schuller, Andrew D. Kent

**Affiliations:** † Center for Quantum Phenomena, Department of Physics, New York University, New York, New York 10003, United States; ‡ Department of Physics, University of California San Diego, La Jolla, California 92093, United States; § Department of Materials Science and Engineering, University of California−Davis, Davis, California 95616, United States; ∥ Department of Physics and Astronomy, University of Denver, Denver, Colorado 80210, United States

**Keywords:** metal−insulator transition (MIT), transition metal oxide, voltage-triggered MIT, spin-torque ferromagnetic resonance, synaptic weights tuning

## Abstract

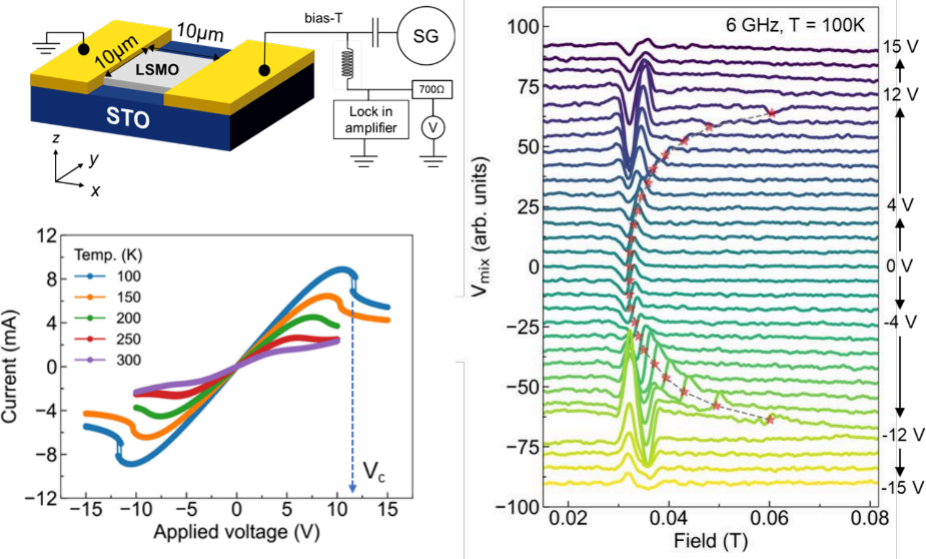

Metal–insulator transitions (MITs) in resistive switching materials can be triggered by an electric stimulus that produces significant changes in the electrical response. When these phases have distinct magnetic characteristics, dramatic changes in the spin excitations are also expected. The transition metal oxide La_0.7_Sr_0.3_MnO_3_ (LSMO) is a ferromagnetic metal at low temperatures and a paramagnetic insulator above room temperature. When LSMO is in its metallic phase, a critical electrical bias has been shown to lead to an MIT that results in the formation of a paramagnetic resistive barrier transverse to the applied electric field. Using spin-transfer ferromagnetic resonance spectroscopy, we show that even for electrical biases less than the critical value that triggers the MIT, there is magnetic phase separation, with the spin-excitation resonances varying systematically with applied bias. Therefore, voltage-triggered MITs in LSMO can alter magnetic resonance characteristics, offering an effective method for tuning synaptic weights in neuromorphic circuits.

Voltage-induced metal–insulator transition (MIT) phenomena are being actively explored for applications in neuromorphic computing.^1−5^ For example, a voltage can drive a volatile *insulator-to-metal* transition in memristive devices by forming conducting filaments parallel to the electric field and current flow, as occurs in VO_2_.^6−12^ These devices have been used to produce spiking behavior in neuromorphic computing.^13−16^ Materials with a *metal-to-insulator* transitions—a metallic state at low temperature and insulating state at high temperature—can show distinct behavior; an applied voltage can lead to switching into a high resistance state by the formation of a resistive barrier transverse to the applied electrical field.^17−20^ Besides MIT switching devices, spintronic devices also show great potential for neuromorphic applications.^21−27^ MIT switching and spintronics are significant, yet independent, approaches to implement hardware-based neurons. Integrating resistive and spintronic functionalities within a single material platform could greatly enhance the capabilities of neuromorphic circuits by harnessing the combined advantages of both effects.^25^

The transition metal oxide La_0.7_Sr_0.3_MnO_3_ (LSMO) demonstrates great potential in this regard, as it is a material with an MIT^28−31^ with a simultaneous magnetic phase transition.^32^ The low-temperature phase of LSMO is a ferromagnetic metal with a Curie temperature of *T*_c_ ≈ 340 K, and its high-temperature phase is a paramagnetic insulator.^28^ Thus, a bias voltage applied to a metallic sample that drives the MIT also produces magnetic phase separation with the formation of a paramagnetic insulating phase between ferromagnetic metallic regions. Previous studies presented that versatile novel phenomena are primarily observed at/above the MIT.^29−31^ Here we show that the magnetic resonance response depends strongly on the applied bias voltage and temperature. Notably, the response splits into multiple well-defined resonances for voltages less than the critical electrical bias (*V*_c_). The spin-torque ferromagnetic resonance (ST-FMR) measurements, therefore, show that magnetic phase separation appears prior to the voltage-driven MIT. Due to the combined MIT switching and spintronic properties in multifunctional LSMO, applying electrical bias provides a sensitive new means to control magnetic resonance characteristics. This, in turn, offers an effective tuning of synaptic weights in spin-oscillators functioning as neurons and spin-resonators functioning as the synapses, which is of great interest for spin-based neuromorphic circuits.^23−25^

A 20 nm thick LSMO thin film (for details, see Supporting Information S1) was patterned into a device with a channel width of 10 μm and distance between the Pd/Au electrical contacts of 10 μm, as illustrated schematically in Figure 1a. Figure 1b shows the resistance versus temperature, which decreases with decreasing temperature below the phase change temperature of *T*_c_ ≈ 340 K. Figure 1c shows the voltage–current characteristics of the device in the ferromagnetic phase at 100, 200, 250, and 300 K. At 300 K, the characteristics are nonlinear yet continuous. However, upon cooling the device below 250 K, a marked voltage-controlled N-type negative differential resistance (NDR)^33^ can be observed. A pronounced jump and hysteretic switching at critical values could be observed at 150 K and below (*V*_c_ = 10 V at 150 K, *V*_c_ = 12 V at 100 K). The NDR indicates that the device resistance increases with increasing bias voltage and the jump in the *I*–*V* curve corresponds to the local phase transition in LSMO producing resistive switching. Prior work on similar samples attributed this switching to localized Joule heating raising the device temperature above *T*_c_.^28^

**Figure 1:**
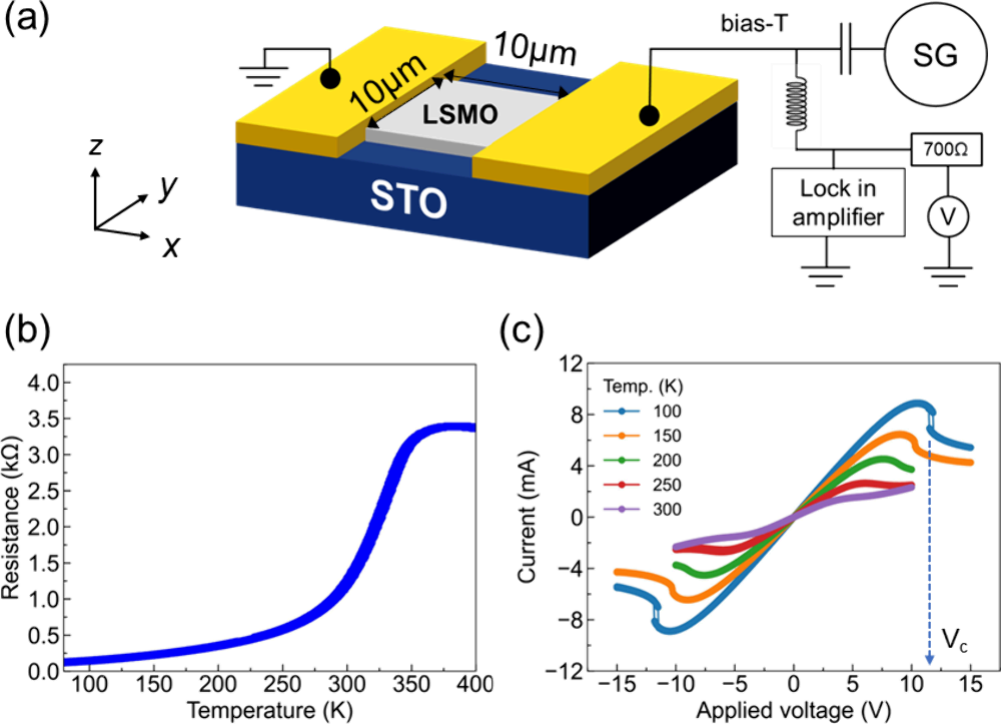
(a) Illustration of measurement setup. The LSMO device has a lateral dimension of 10 μm by 10 μm. (b) LSMO device resistance as a function of the temperature. (c) *I*–*V* curve measurements at various temperatures. *V*_c_ indicates the critical voltage at 100 K.

ST-FMR measurements were conducted on the device at 100 K using the setup illustrated in Figure 1a, which included a DC voltage source connected via a bias tee (for details, see Supporting Information S1). This configuration allows us to record the magnetic response under applied DC biases. Initially, we investigated the ST-FMR with zero DC bias applied to the sample, as shown in Figure 2a. The ST-FMR signal could be fit by a superposition of symmetric and antisymmetric Lorentzian functions,^34^ as shown in Figure 2b (for details see Supporting Information S2). This analysis gives the resonance field and resonance line width. In Figure 2c the line widths (Δ*H*) are plotted as a function of frequency. The damping constant (α) and inhomogeneous line width (Δ*H*_0_) are determined using the relation Δ*H* = Δ*H*_0_ + 2πα*f*/γ, where *f* is the frequency and γ is the gyromagnetic ratio.^35^ The damping constant, 0.0045, and the inhomogeneous line width, 0.47 mT, are of the same order as those of previous reports for LSMO thin films.^36,37^Figure 2(d) shows the frequency versus the resonance field. This data is fit to the Kittel model^38^
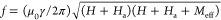
to give an effective magnetization *M*_eff_ of 0.96 T and anisotropy field *H*_a_ of 13.8 mT. ST-FMR was also conducted as a function of the temperature, and the inset shows how the effective magnetization depends on temperature. The decrease in effective magnetization with increasing temperature is expected for a ferromagnet approaching the Curie temperature. Overall, high effective magnetization, low damping, and narrow inhomogeneous line width indicate the structural homogeneity and high crystalline quality of our LSMO sample.

**Figure 2:**
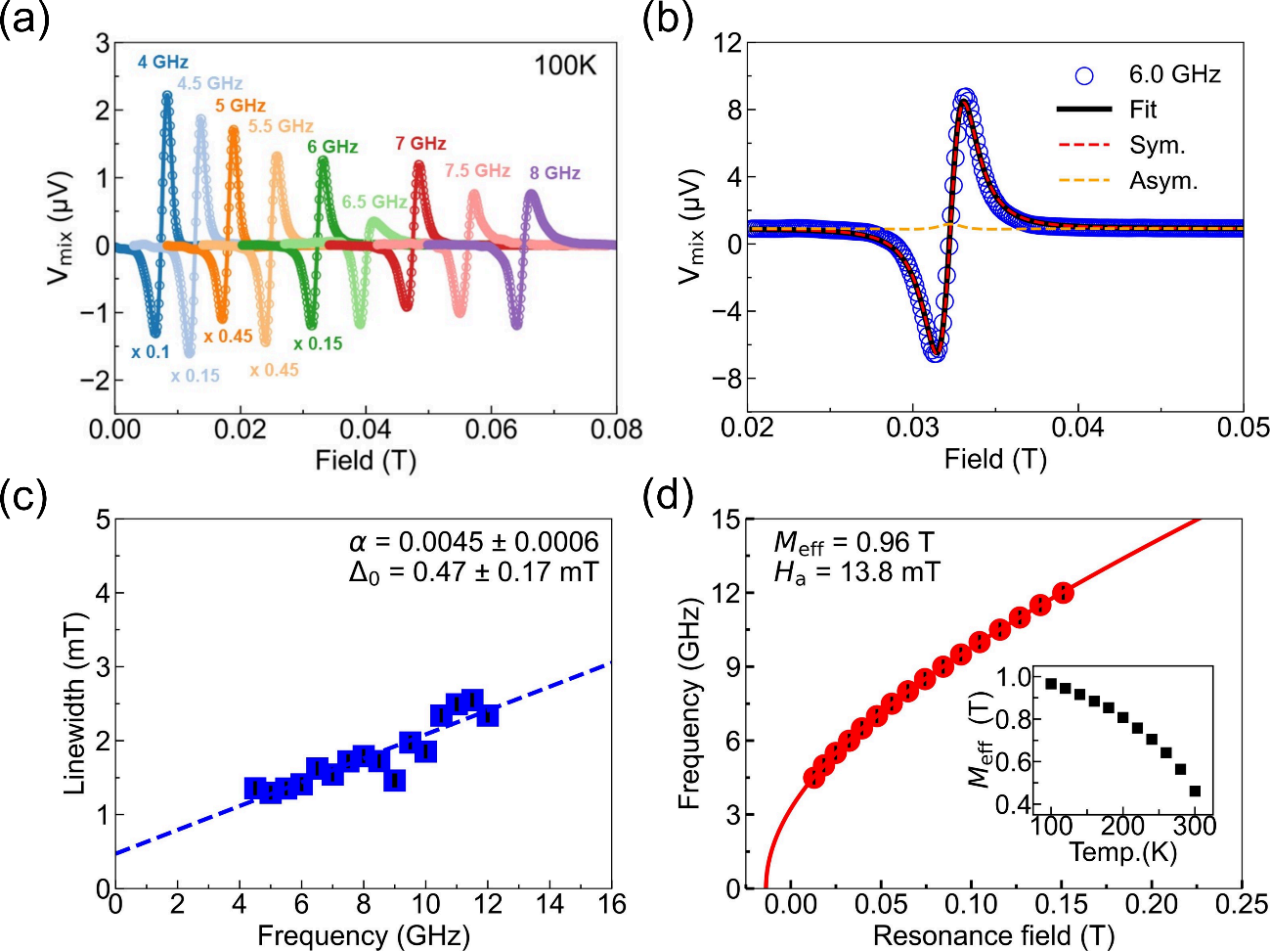
(a) ST-FMR resonance response at 100 K, for frequencies from 4 to 8 GHz at zero DC bias voltage. Several curves have been scaled as indicated. (b) The 6 GHz resonance line fit to the derivative of Lorentzian functions characterizing the symmetric (red) and asymmetric components (yellow) response. (c) ST-FMR line width as a function of frequency with a linear fit that enables determination of the damping constant α and inhomogeneous line width Δ*H*_0_. (d) The resonant field as a function of frequency. Inset: effective magnetization as a function of temperature. The error bars for the line widths and the resonance fields are indicated as black lines and are within the size of the data points.

We used ST-FMR to determine the magnetic properties as a function of the bias voltage at 100 K. The results are shown in Figure 3a. The applied voltage varied from −15 to +15 V to cover the range in which the voltage-driven phase transition and NDR are observed. Within the voltage range −3 to +3 V the ST-FMR spectrum exhibits a single Lorentzian peak. The peak shifts as the voltage increases, which is attributed to Joule heating. As the applied voltage reaches ±4 V, the line shape resembling a single peak can no longer be fit with a Lorentzian function. Upon increasing the voltage to ±6 V, we observed that the single peak splits into three distinct peaks with the rightmost peak moving to higher field while the two lower field peaks remain nearly at fixed fields. When the applied voltage reaches the critical value of ±12 V, the higher field peak is no longer visible. The multiple peaks indicate that there are multiple magnetic phases. From previous measurements, however, phase separation is not expected to emerge at voltage below *V*_c_.^28^ Even above *V*_c_, the expected phase separation is between the ferromagnetic matrix and the paramagnetic barrier, which cannot account for the observed multiple ST-FMR peaks. We note that the same peak separation behavior was found in multiple samples (see Supporting Information S3).

**Figure 3:**
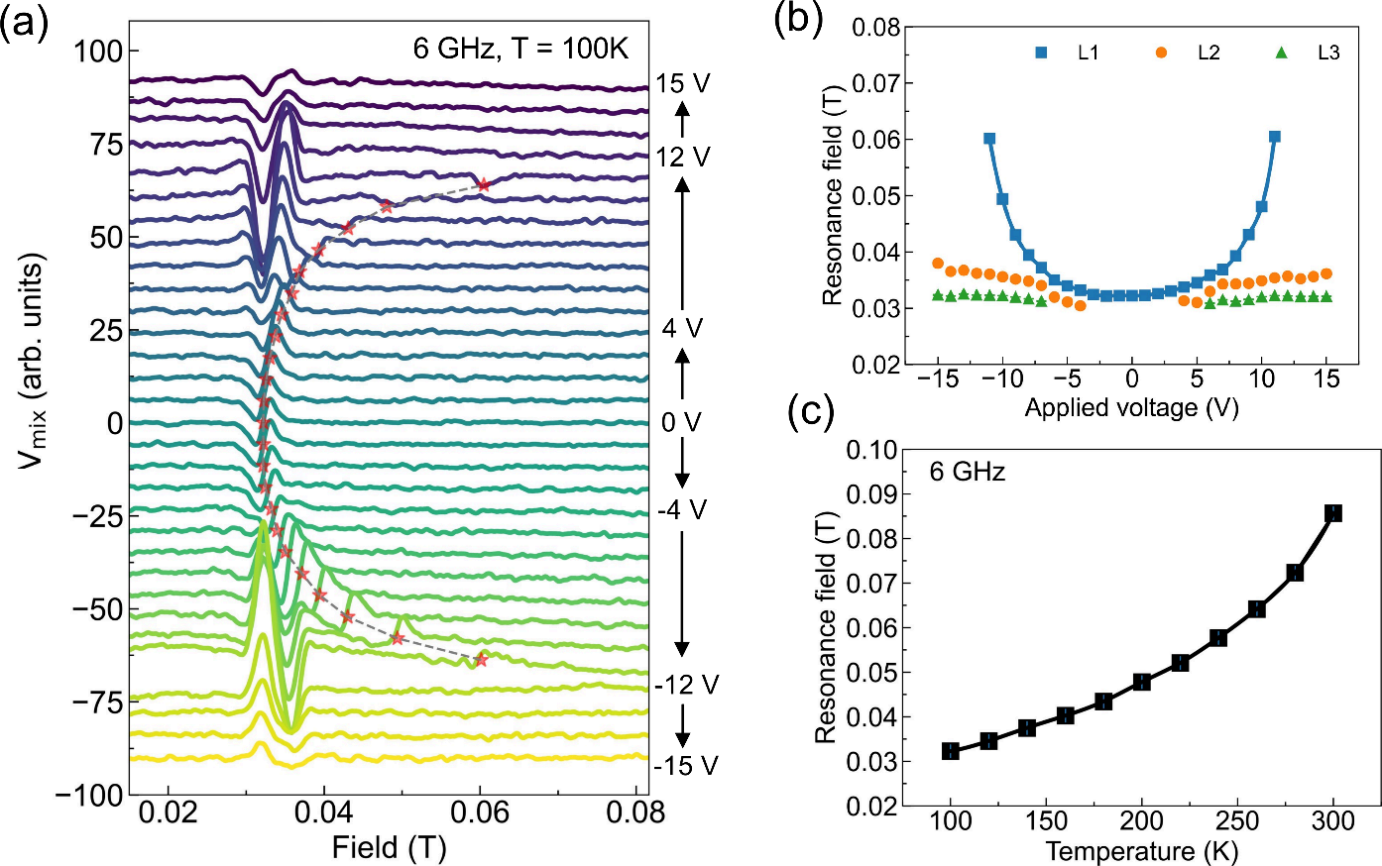
(a) ST-FMR signal as a function of bias voltage from −15 to +15 V at 6 GHz and 100 K. Here, *V*_mix_ represents the signal collected by the lock-in amplifier. The data has been shifted vertically for clarity. (b) Summary of resonant fields L1, L2, and L3 as a function of the applied bias voltage at 6 GHz and 100 K. L1, L2, and L3 represent the resonance peaks in order of appearance as the applied voltage changes (details in Supporting Information S5). (c) Resonance field at 6 GHz as a function of temperature at zero bias voltage. The error bars for the resonance fields are very small and fall within the size of the data points.

To analyze the data, we label the peaks *in order of appearance* as a function of the applied voltage L1, L2, and L3 (for details on the data analysis refer to Supporting Information S4 and S5). These resonance fields are plotted in Figure 3b. The L1 peak exhibits significant sensitivity to changes in applied voltage, whereas the L2 and L3 peaks show comparatively less change. According to the Kittel resonance condition, for a fixed RF frequency, a higher resonance field indicates a lower effective magnetization. Consequently, under applied voltages, peak L1 is indicative of parts of the device that experience heating that reduces the effective magnetization. In contrast, the L2 and L3 peaks must characterize regions that remain at a relatively lower temperature. The clear peak separation thus shows that regions with distinct magnetic properties form in response to the voltage bias. To be quantitative, we conducted measurements of the resonance field versus temperature at 6 GHz at zero voltage bias, which are shown in Figure 3c. By comparing the L1 resonance fields with the resonance field at zero bias versus temperature, we infer that resonance L1 is consistent with a region that is at 250 K when the applied bias is 11 V, while the area of L2 experiences a moderate temperature increase to 160 K. In contrast, the L3 resonance is linked to a sample region that is not significantly heated.

Previous research demonstrated that in the voltage-triggered MIT of LSMO a resistive paramagnetic barrier spontaneously forms transverse to the applied electric field and expands as the voltage increases. Such a phase separation was previously observed only above the critical voltage.^28^ Our work, however, reveals that bias voltage can induce multiple well-defined magnetic resonances *before* the bias reaches the critical value. This result suggests distinct nonuniform magnetic properties within the LSMO sample because at equilibrium a single ferromagnetic layer with a homogeneous magnetic properties and temperature distribution exhibits only one resonance peak (see Supporting Information S6).

We propose a simple model to explain the experimental observations that are illustrated schematically in Figure 4. At zero voltage, as shown in Figure 4a, the LSMO device has a uniform temperature and magnetization distribution and, thus, a single ST-FMR resonance. With the applied voltage, the sample heats initially without apparently inducing a significant temperature gradient or variation in magnetic properties (Figure 4b). The ST-FMR resonance peak shifts to higher field, indicating a heating-induced decreased magnetization. As illustrated in Figure 4c, with increasing applied voltage there is an instability: a local region heats, leading to an increase in its resistance and increased power dissipation in that region. This increased local power dissipation causes the local region to heat further; i.e., this is a positive feedback loop in which heating of a device region causes more heating of that region. Within this model, this positive feedback eventually leads to the formation of a paramagnetic insulating barrier when the voltage bias exceeds *V*_c_, as shown in Figure 4d. As in the prior work,^28^ it seems reasonable to assume that the hot region is close to the middle of the LSMO device, the region furthest from the high thermal conductivity electrical contacts. However, it is also possible that the phase separation starts at a structural inhomogeneity, a locally more resistive sample region, or sample defects.

**Figure 4:**
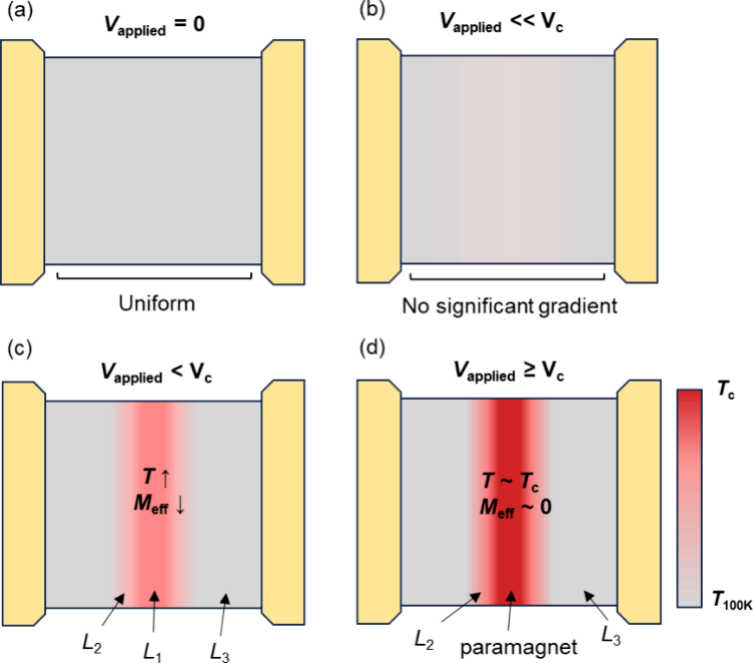
Schematic illustration of the proposed voltage-induced magnetic phase separation mechanism. (a) *V* = 0 V. The whole sample has a uniform temperature and effective magnetization. (b) *V* ≪ *V*_c_. The entire sample heats but without a significant temperature gradient. (c) *V* < *V*_c_. The center area is heated up by the applied voltage and forms a hot region with lower effective magnetization, whereas the side areas show a relatively lower constant temperature. (d) *V* ≥ *V*_c_. When the device region reaches the critical temperature, a paramagnetic resistive barrier forms where the effective magnetization drops to zero.

Within this model, before reaching the critical voltage, the device has three distinct temperature zones: the hottest area, an intermediate region, and an area that remains cool, consistent with the three observed ST-FMR resonances. Upon reaching the critical voltage, the area with the highest temperature transitions into a paramagnetic state, causing its resonance peak to vanish, while the cooler areas continue to exhibit ferromagnetic properties. Therefore, only two magnetic resonances can be detected above the critical voltage. We note that this situation is distinct from typical scenarios in condensed matter physics in which materials have imperfections or are spatially inhomogeneous leading to district magnetic resonances associated with material nonuniformities. Here the formation of multiple resonances is an intrinsic characteristic associated with electric-field-induced phase separation. Our experimental results reveal a three-temperature zone during voltage application, highlighting the complexity of MIT in LSMO and suggesting that factors beyond resistance and temperature, such as strain and doping effects, need to be considered in future theoretical studies.

In conclusion, we found unusual behavior of the ST-FMR on LSMO microstructures, indicating the presence of intrinsic voltage-induced phase separation. The single well-defined resonance at low voltages splits into multiple resonances even when the applied voltage is below the critical voltage, which further induces magnetic phase separation, leading to the formation of a paramagnetic insulating barrier at the critical voltage. In spin oscillator-based neuron networks, the voltage control of individual oscillator frequencies is a major challenge; e.g., electrical fields only lead to small changes in the magnetic resonance characteristics of ferromagnetic transition metals. The large electrically tunable magnetic resonance in LSMO can thus provide a means to tune synapses or spin-oscillator neurons in the spintronic neural network.^25^ LSMO is therefore a material that can be used both for MIT switching and for spintronic applications, offering new possibilities for spintronic neuromorphic devices.

## Supporting Information Available

Methods, ST-FMR signal analysis, additional devices, voltage-dependent ST-FMR signal processing, multipeak ST-FMR data fitting, and temperature-dependent ST-FMR spectra (PDF)
