# Supplementary material for: Electrical Control of Magnetic Resonance in Phase Change Materials
Source: Nano Lett. 2024 Sep 4;24(37):11476–81. doi: 10.1021/acs.nanolett.4c02697 (PMC11421091; doi:10.1021/acs.nanolett.4c02697)
Supplement: Supplementary file 1 — nl4c02697_si_001.pdf [file nl4c02697_si_001.pdf]

Electrical control of magnetic resonance in phase change materials

Tian-Yue Chen[1*], Haowen Ren[1], Nareg Ghazikhanian[2], Ralph El Hage[2], Dayne Y. Sasaki[3], Pavel Salev[4], Yayoi Takamura[3], Ivan K. Schuller[2], and Andrew D. Kent[1*]

[1]Center for Quantum Phenomena, Department of Physics, New York University, New York, NY 10003, USA

[2]Department of Physics, University of California San Diego, La Jolla, CA 92093, USA

[3]Department of Materials Science and Engineering, University of California – Davis, Davis, California 95616, USA

[4]Department of Physics and Astronomy, University of Denver, Denver, Colorado 80210, USA

*Email: Tian-Yue Chen: tc3836@nyu.edu, Andrew D. Kent: andy.kent@nyu.edu

Supplementary materials

S1. Methods

S2. Analysis of ST-FMR signal characteristics

S3. Additional devices

S4. Voltage-dependent ST-FMR signal processing

S5. Multi-peak fitting

S6. Temperature dependent ST-FMR spectrum

S1. Methods

A 20 nm thick layer of $La_{0.7}Sr_{0.3}MnO_3$ (LSMO) was epitaxially grown on a (001)-oriented $SrTiO_3$ (STO) substrate by pulsed laser deposition as described in Ref. 23. The film was patterned into 10 µm by 10 µm micron-sized features with Pd/Au electrical contacts for electrical transport and ST-FMR measurements. ST-FMR measurements were performed in the temperature range 100 to 300 K with voltage applied with a Keithley 2400 source meter. The modulation frequency of the magnetic field is 433 Hz. A 700 Ω resistor in series is to manipulate the critical voltage and initial barrier size. All measurements were conducted using a Quantum Design Physical Property Measurement System (PPMS). ST-FMR was conducted with an Anritsu MG3692B RF signal generator in the 4 to 12 GHz frequency range. Magnetic field was applied in plane at 45 degrees to the RF current to obtain a large signal.

S2. Analysis of ST-FMR signal characteristics

The measured field modulated ST-FMR[1-2] signal is described by the derivative of a Lorentzian function:

$$V(B) = \frac{-S\,(B\text{-}B_0)\Delta B + A\,((B\text{-}B_0)^2 - (\Delta B/2)^2)}{((B\text{-}B_0)^2 - (\Delta B/2)^2)^2} \quad ,$$

where S, A, B_0, B, ΔB are the symmetric component, the asymmetric component, the resonance field, the external field in a vacuum (i.e. $B = \mu_0 H$, where μ_0 is the permeability of free space) and the linewidth, respectively.

The Lorentzian fit to our data, shows a symmetric component in the LSMO, which could originate from either the self-generated spin-orbit torque in LSMO[3] or from signals associated with Oersted fields associated with displacement currents in the substrate[4].

S3. Additional devices

To ensure the reproducibility of our findings, we conducted voltage-dependent ST-FMR on various devices and they all showed the same voltage-induced resonance peak separation below their critical biases. Here, we showed two more ST-FMR spectrum from device #2 and device #3 in Figure S1(a) and (b). Both devices have critical voltages of 11 V.

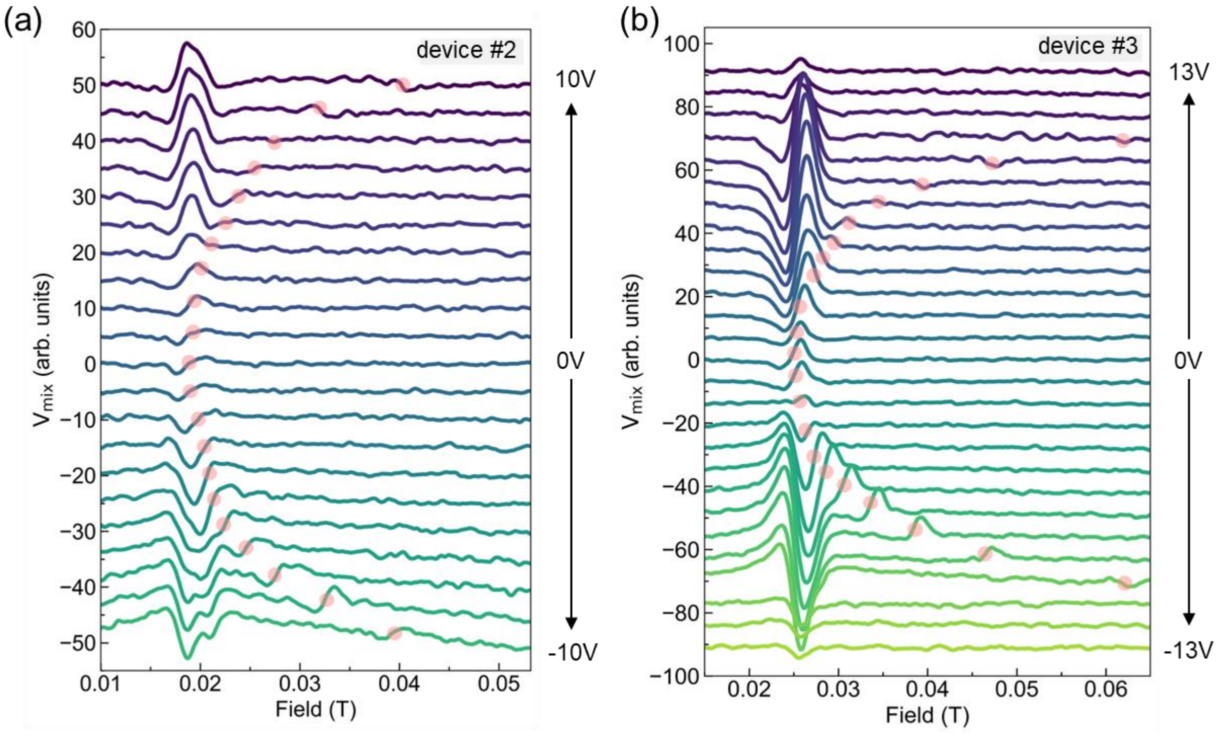

Figure S1. Voltage-dependent ST-FMR spectrum from (a) device #2 at 5 GHz and (b) device #3 at 5.5 GHz. Both measurements were carried out at 100 K.

S4. Voltage-dependent ST-FMR signal processing

In the voltage dependent measurement, a background signal can be found which is known to affect the peak shape analysis[5]. As seen in the raw data, the background signal follows a polynomial curve as a function of the applied field, becoming significant at low field. To mitigate the effect of the background we selected a frequency of 6 GHz to ensure a moderate resonance field and peak intensity with good signal to noise. To perform the Lorentzian function fitting, we removed a polynomial background signal in the raw data and fitted the subtracted data, as shown in Figure S2. We excluded data points in the range around the resonance peak in the polynomial fit of the background. For consistency, the fitting range covers all three peaks within the entire sweeping field, and we note that the fitting range has no appreciable variation with applied voltages. However, it is important to note that the resonance field we extract from the fit is not sensitive to the background.

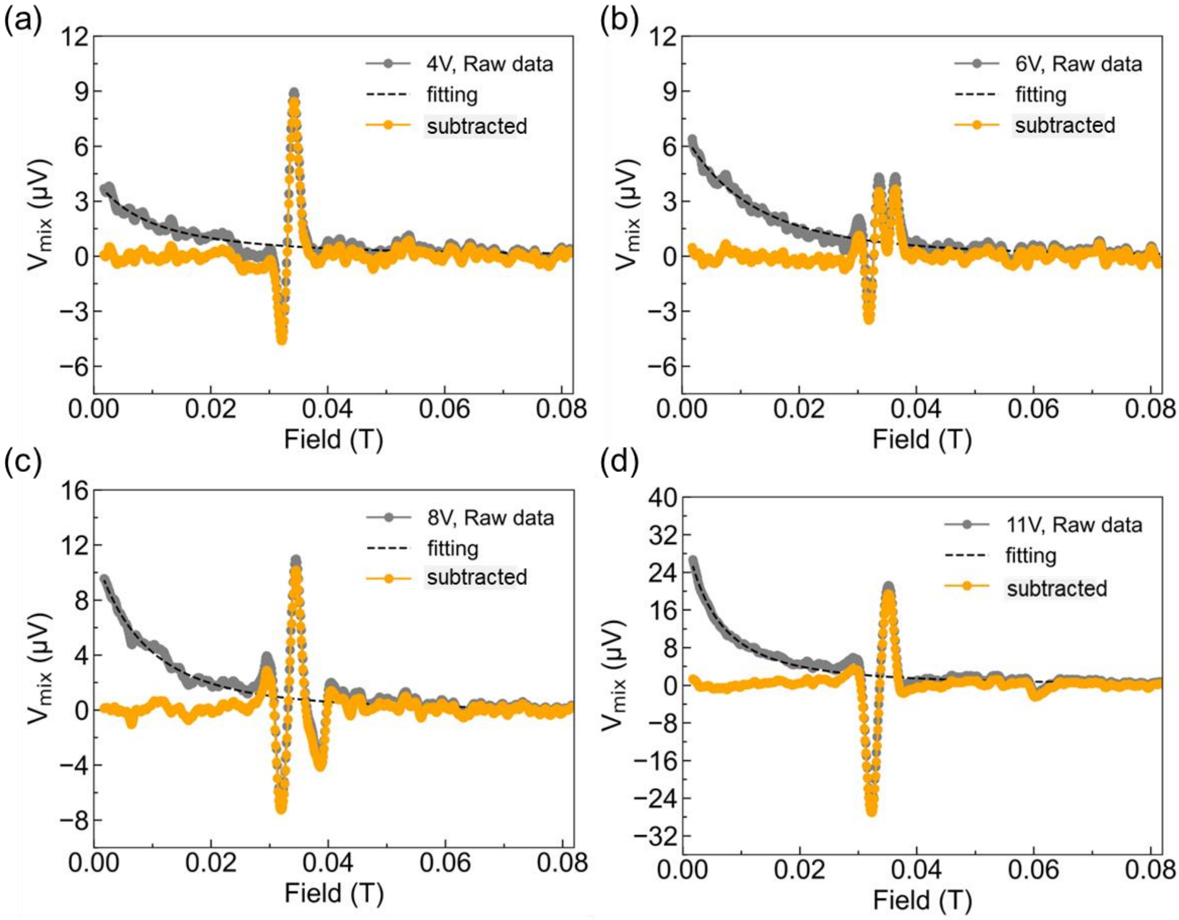

Figure S2. Data processing. Representative data fitting for dc voltage biases of (a) 4 V, (b) 6 V, (c) 8 V, and (d) 11 V. The gray points are the raw data, the dashed line is a fit to the background signal and the yellow points are the data used in the Lorentzian fit to determine the resonance field positions and linewidths.

S5. Multi-peak fitting

As the voltage increases, the ST-FMR line shape changes and a single Lorentzian function cannot describe the measured data. Therefore, we included multiple Lorentzian functions in the fit. With increasing applied voltage, three peaks appear so three identical Lorentzian functions were introduced to characterize the three resonances:

$$L_1(B) = \frac{-S_1\,(B-B_{0,1})\Delta B_1 + A_1\,((B-B_{0,1})^2 - (\Delta B_1/2)^2)}{((B-B_{0,1})^2 - (\Delta B_1/2)^2)^2}$$

$$L_2(B) = \frac{-S_2\ (B\text{-}B_{0,2})\Delta B_2 + A_2\ ((B\text{-}B_{0,2})^2 - (\Delta B_2/2)^2)}{((B\text{-}B_{0,2})^2 - (\Delta B_2/2)^2)^2}$$

$$L_3(B) = \frac{-S_3\ (B\text{-}B_{0,3})\Delta B_3 + A_3\ ((B\text{-}B_{0,3})^2 - (\Delta B_3/2)^2)}{((B\text{-}B_{0,3})^2 - (\Delta B_3/2)^2)^2}$$

Within -3 V to 3 V, the ST-FMR lineshape can be accurately modeled with a single Lorentzian function, denoted as L1. Starting at 5 V, a second Lorentzian function (L2) needs to be included in the fitting equation, which is represented as $V(B) = L_1(B) + L_2(B)$. Upon increasing the voltage to 8 V, a third peak (L3) emerges, leading us to use a three-peak equation $V(B) = L_1(B) + L_2(B) + L_3(B)$ for data analysis. Above the critical voltage ($V \geq 12$ V), the L1 peak disappears, leaving only the L2 and L3 peaks, resulting in the equation $V(B) = L_2(B) + L_3(B)$. These fitting functions enable a precise representation of the experimental data. Figure S3 displays the multi-peak fitting for the measured data points.

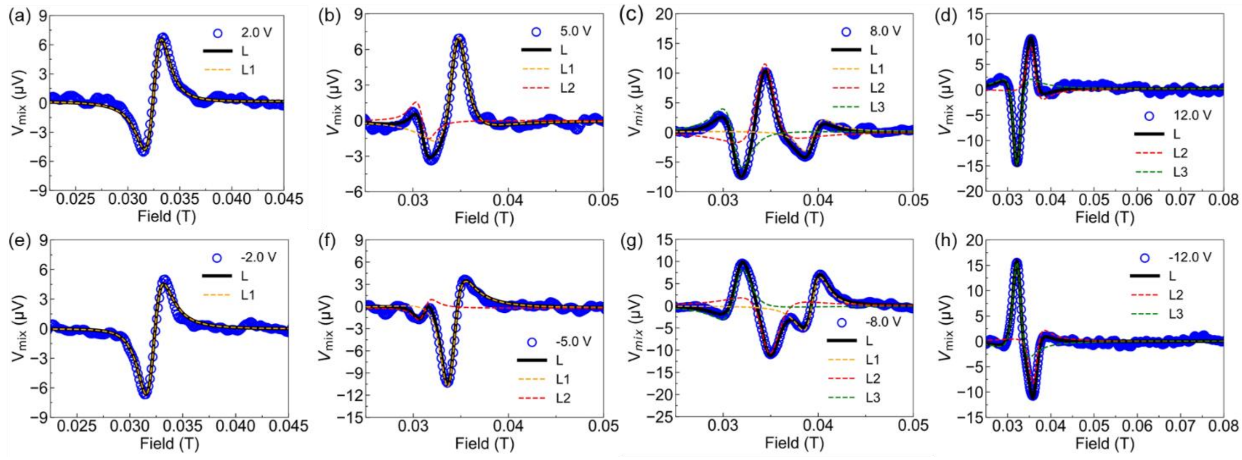

Figure S3. Multi-Lorentzian function fitting. ST-FMR spectrum at (a) 2 V, (b) 5 V, (c) 8 V, (d) 12 V, (e) -2 V, (f) -5 V, (g) -8 V, and (h) -12 V.

S6. Temperature dependent ST-FMR spectrum

In this discussion, we explore alternative origins for the appearance of multiple resonance peaks. Assuming the sample undergoes heating with a smooth variation in temperature below the critical voltage one would expect the ST-FMR signal to exhibit a single response with a linewidth that broadens with increasing voltage, rather than presenting sharp, well-defined spectra. To check this, we measured the ST-FMR spectra at 6 GHz at 120 K, 180 K, 220 K,

and 260 K at zero bias voltage as shown in Figure S4. All lines fit well with a single Lorentzian profile without the need of multiple distinct peaks.

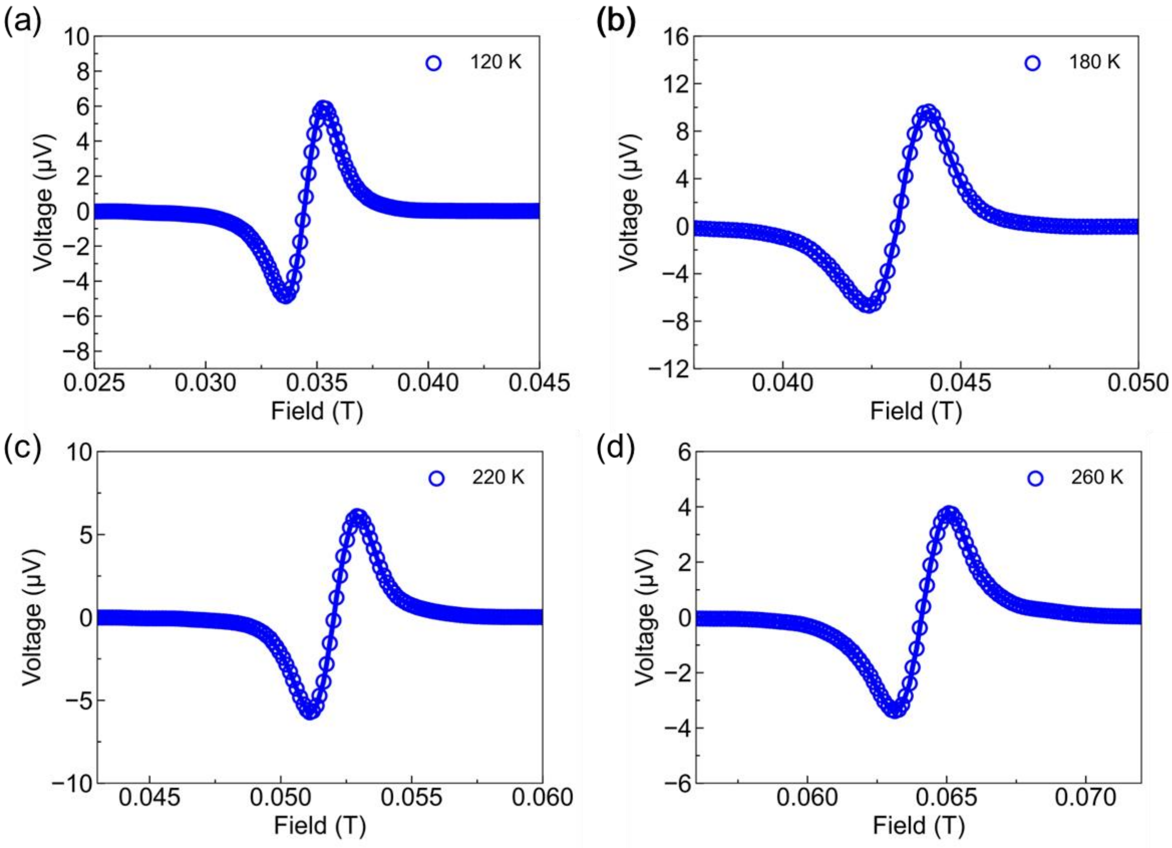

Figure S4. 6 GHz ST-FMR spectra at (a) 120 K, (b) 180 K, (c) 220 K, and (d) 260 K and zero DC bias voltage.

References

1. Xu, J.-W.; Kent, A. D., Charge-To-Spin Conversion Efficiency in Ferromagnetic Nanowires by Spin Torque Ferromagnetic Resonance: Reconciling Lineshape and Linewidth Analysis Methods. *Physical Review Applied* **2020,** *14* (1), 014012.

2. Gonçalves, A. M.; Barsukov, I.; Chen, Y.-J.; Yang, L.; Katine, J. A.; Krivorotov, I. N., Spin torque ferromagnetic resonance with magnetic field modulation. *Applied Physics Letters* **2013,** *103* (17).

3. Gupta, P.; Park, I. J.; Swain, A.; Mishra, A.; Amin, V. P.; Bedanta, S., Self-induced inverse spin Hall effect in $La_{0.67}Sr_{0.33}MnO_3$ films. *Physical Review B* **2024,** *109* (1), 014437.

4. Jiang, D.; Chen, H.; Ji, G.; Chai, Y.; Zhang, C.; Liang, Y.; Liu, J.; Skowroński, W.; Yu, P.; Yi, D.; Nan, T., Substrate-induced spin-torque-like signal in spin-torque ferromagnetic resonance measurement. *Physical Review Applied* **2024,** *21* (2), 024021.

5. Karimeddiny, S.; Ralph, D. C., Resolving Discrepancies in Spin-Torque Ferromagnetic Resonance Measurements: Lineshape versus Linewidth Analyses. *Physical Review Applied* **2021,** *15* (6), 064017.
